# Linking ABCC6 Deficiency in Primary Human Dermal Fibroblasts of PXE Patients to p21-Mediated Premature Cellular Senescence and the Development of a Proinflammatory Secretory Phenotype

**DOI:** 10.3390/ijms21249665

**Published:** 2020-12-18

**Authors:** Janina Tiemann, Thomas Wagner, Christopher Lindenkamp, Ricarda Plümers, Isabel Faust, Cornelius Knabbe, Doris Hendig

**Affiliations:** Institut für Laboratoriums-und Transfusionsmedizin, Herz-und Diabeteszentrum Nordrhein-Westfalen, Universitätsklinik der Ruhr-Universität Bochum, 32545 Bad Oeynhausen, Germany; jtiemann@hdz-nrw.de (J.T.); thomas.wagner01@gmx.de (T.W.); clindenkamp@hdz-nrw.de (C.L.); rpluemers@hdz-nrw.de (R.P.); ifaust@hdz-nrw.de (I.F.); cknabbe@hdz-nrw.de (C.K.)

**Keywords:** pseudoxanthoma elasticum, cellular senescence, senescence-associated secretory phenotype

## Abstract

Pseudoxanthoma elasticum (PXE) is a rare autosomal-recessive disorder that is mainly caused by mutations in the *ATP-binding cassette sub-family C member 6* (*ABCC6*) gene. Clinically PXE is characterized by a loss of skin elasticity, arteriosclerosis or visual impairments. It also shares some molecular characteristics with known premature aging syndromes like the Hutchinson–Gilford progeria syndrome (HGPS). However, little is known about accelerated aging processes, especially on a cellular level for PXE now. Therefore, this study was performed to reveal a potential connection between premature cellular aging and PXE pathogenesis by analyzing cellular senescence, a corresponding secretory phenotype and relevant factors of the cell cycle control in primary human dermal fibroblasts of PXE patients. Here, we could show an increased senescence-associated β-galactosidase (SA-β-Gal) activity as well as an increased expression of proinflammatory factors of a senescence-associated secretory phenotype (SASP) like interleukin 6 (IL6) and monocyte chemoattractant protein-1 (MCP1). We further observed an increased gene expression of the cyclin-dependent kinase inhibitor (CDKI) *p21*, but no simultaneous induction of *p53* gene expression. These data indicate that PXE is associated with premature cellular senescence, which is possibly triggered by a p53-independent p21-mediated mechanism leading to a proinflammatory secretory phenotype.

## 1. Introduction

Pseudoxanthoma elasticum (PXE) is a rare autosomal-recessive disorder that is characterized by calcification and fragmentation of elastic fibers. This leads to ectopic mineralization of connective tissue, causing a loss of skin elasticity with strong wrinkle formation, arteriosclerosis and the development of angioid streaks, which can trigger choroidal neovascularization, possibly ending up in blindness [1]. The underlying genetic defects are mutations in the *ATP-binding cassette sub-family C member 6* (*ABCC6*) gene resulting in a deficiency of the encoded ABC-transporter protein. *ABCC6* is mainly expressed in the liver and just to a lesser extent in peripheral, but primarily affected tissues. Although over 300 possible PXE-causing *ABCC6* mutations are revealed by now, the physiological substrate of the *ABCC6* transporter is still unknown [1,2,3,4].

However, studies revealed that PXE shares some molecular characteristics with other heritable diseases like the Hutchinson–Gilford progeria syndrome (HGPS). Thus, HGPS and PXE are characterized by a disturbed pyrophosphate (PPi) homeostasis with an increase in alkaline phosphatase activity and decreased ATP and PPi plasma levels leading to calcification processes in both cases [5,6,7]. Furthermore, it was shown that therapy with statins and bisphosphonates could be advantageous for PXE as well as HGPS patients. As the pathogenesis of HGPS is comparatively well-known, and patients clearly show accelerated aging symptoms, HGPS is classified as a premature aging syndrome [7,8,9,10]. Those accelerated aging processes in HGPS patients are promoted to a considerable extent by an increase in cellular senescence due to the inherited genetic defect [11]. Cellular senescence itself is categorized as a hallmark of aging and is, thus, further defined as a permanent growth arrest of aged and/or damaged cells to avoid further spreading of the cellular damage or malignant degeneration. As a logical consequence, the amount of senescent cells increases during the course of aging as cellular damages accumulate stronger and/or the clearance of senescent cells due to an attenuated immune response is decreased. Besides the positive effect of tumor suppression, senescent cells also provoke destructive effects as an accumulation of senescent cells can induce new or further promote existing pathologies because those cells differ in expression and secretion profiles from proliferating cells. These differences include, e.g., insoluble factors and proteins as chemokines, growth factors and proteases, which modulate the tissue microenvironment and affect cellular communication in an autocrine or paracrine manner [12].

With respect to the similarities between HGPS and PXE, several known molecular characteristics of PXE could possibly be connected to aging and cellular senescence but are barely discussed or intensively evaluated against this background. Thus, senescent cells can, e.g., accelerate wound healing by inducing myofibroblast differentiation [13]. Faust et al. demonstrated a faster wound healing of PXE fibroblasts compared to normal human dermal fibroblasts (NHDF) in vitro [14]. Studies of Diekmann et al. showed increased levels of the matrix metalloproteinases (MMP) -2 and MMP-9 in PXE patients [15]. MMPs often contribute to the secretory phenotype of senescent cells and are also associated with choroidal neovascularization, a characteristic of age-related macular degeneration. Choroidal neovascularization is also a clinical manifestation seen for PXE patients [12,16,17]. Additionally, a study of Miglionico et al. showed an increase in cellular senescence in stable *ABCC6* knockdown HepG2 clones, probably by induction of reductive stress and p21 expression [18]. However, hepatocytes indeed express high levels of *ABCC6* under healthy conditions but are not primarily affected by PXE [19]. Thus, it raises the question of whether a potential increase in the number of senescent cells and, therefore, possible accelerated aging processes in peripheral tissues of PXE patients play a role in PXE pathogenesis, although *ABCC6* is just barely expressed in these tissues anyway. Unfortunately, little is known about the proliferative status and a corresponding secretory phenotype of cells in peripheral tissues of PXE patients. Thus, the evaluation of a potential senescent phenotype of primary human dermal fibroblasts of PXE patients could contribute to the understanding of the molecular PXE pathogenesis within affected peripheral tissues. As it is already known that lipid metabolism plays an important role in PXE pathogenesis [20], the cultivation of the primary human dermal fibroblasts of PXE patients in a medium with lipoprotein depleted fetal calf serum (LPDS) probably further reveals effects that would be otherwise potentially covert by an excess of exogenous lipoproteins when conventional fetal calf serum (FCS) is used. By analyzing key factors of a senescence phenotype, we uncovered a probably direct effect of an *ABCC6* deficiency on the proliferative status and a corresponding secretory phenotype of affected cells in peripheral tissues.

## 2. Results

### 2.1. Altered Cell Morphology and Increased Senescence-Associated-β-Gal (SA-β-Gal) Activity

**Figure 1:**
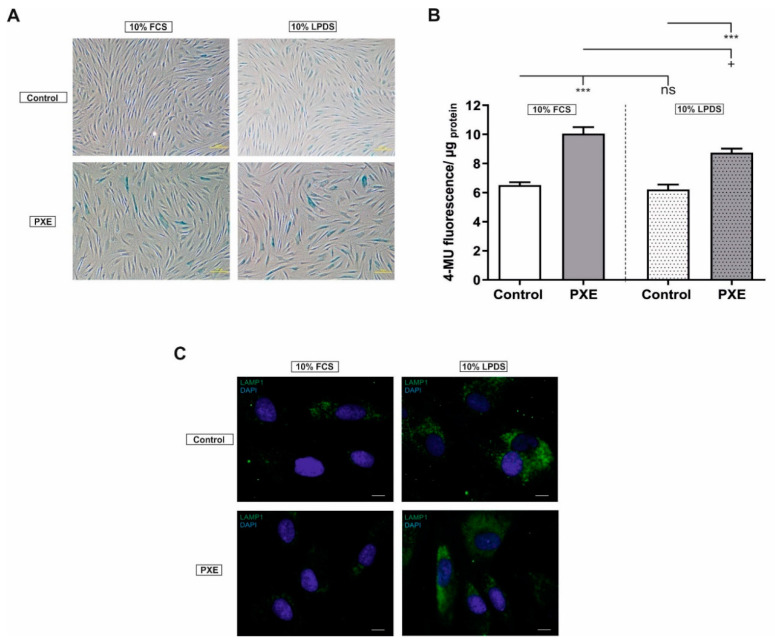
SA-β-Gal activity and immunofluorescence microscopy of LAMP1 for pseudoxanthoma elasticum (PXE) fibroblasts and normal human dermal fibroblasts (NHDF). Fibroblasts were cultivated for 72 h in medium with 10% fetal calf serum (FCS) or 10% lipoprotein-deficient fetal calf serum (LPDS). (**A**) Qualitative senescence assay for PXE fibroblasts (*n* = 3) and NHDF (*n* = 3). Representative images are shown. Scale bar: 100 μm. (**B**) Quantitative senescence assay for PXE fibroblasts (gray, *n* = 3) and NHDF (white, *n* = 3). Data are shown as mean ± SEM. Control/ PXE: *** *p* ≤ 0.001. 10% FCS/10% LPDS: + *p* ≤ 0.05; ns *p* > 0.05. (**C**) Immunofluorescence microscopy of LAMP1 (green) for PXE fibroblasts (*n* = 3) and NHDF (*n* = 3). Cell nuclei were counterstained with DAPI (blue). Representative images are shown. Scale bar: 10 µm.

Prominent factors of cellular senescence are an altered cell morphology and an increased SA-β-Gal activity. As seen in Figure 1A, PXE fibroblasts showed an aberrant cell morphology compared to NHDF when cultivated in medium with 10% FCS as well as in medium with 10% LPDS. Cell morphology of PXE fibroblasts changed from elongated shape to flattened morphology. In addition to this, PXE fibroblasts showed increased staining for SA-β-Gal activity compared to NHDF in both media. As shown in Figure 1B fluorescence signal of SA-β-Gal activity increased significantly in PXE fibroblasts in medium supplemented with 10% FCS (control: 6.52 ± 0.20, PXE: 10.05 ± 0.45; *p* ≤ 0.001) as well as in medium supplemented with 10% LPDS (control: 6.21 ± 0.34, PXE: 8.75 ± 0.28; *p* ≤ 0.001). Furthermore, fluorescence signal for SA-β-Gal activity was significantly decreased in PXE fibroblasts cultivated in 10% LPDS compared to PXE fibroblasts cultivated in 10% FCS (PXE in 10% FCS: 10.06 ± 0.45, PXE in 10% LPDS: 8.75 ± 0.28; *p* ≤ 0.05). No changes were observed between NHDF in both media.

An increase in SA-β-Gal activity is often associated with a change in lysosomal mass or an increase in β-galactosidase activity or lysosomal protein concentration. Thus, we analyzed lysosomal-associated membrane protein (LAMP) 1, a lysosomal protein, to evaluate a potential accumulation of lysosomes. An increase in LAMP1 was detected for PXE fibroblasts as well as for NHDF when cultivated in medium with 10% LPDS compared to cultivation in medium with 10% FCS (Figure 1C). No significant changes for LAMP1 were seen between PXE fibroblasts and NHDF.

### 2.2. Aberrant Lamin B1 (LMNB1) Gene, but No Changes in Protein Expression

**Figure 2:**
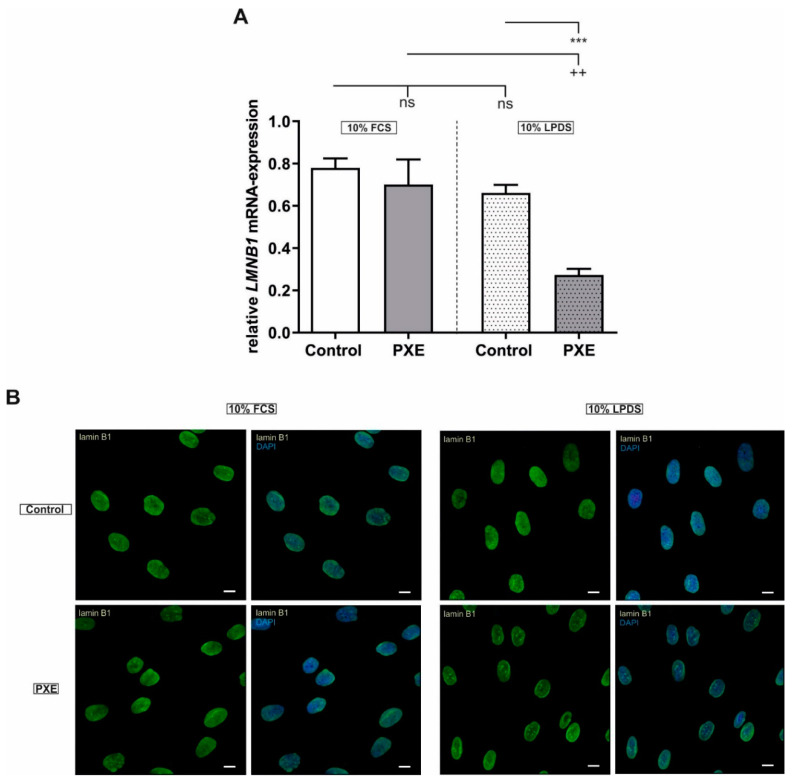
Relative *LMNB1* mRNA-expression and immunofluorescence microscopy of lamin B1 for PXE fibroblasts and NHDF (**A**) Relative *LMNB1* mRNA-expression of PXE fibroblasts (gray, *n* = 3) and NHDF (white, *n* = 3) after 24 h cultivation in 10% FCS or 10% LPDS. Data are shown as mean ± SEM. Control/PXE: *** *p* ≤ 0.001; ns *p* > 0.05. 10% FCS/ 10% LPDS: ++ *p* ≤ 0.01; ns *p* > 0.05. (**B**) Immunofluorescence microscopy of lamin B1 (green) for PXE fibroblasts (*n* = 3) and NHDF (*n* = 3) after 72 h cultivation in 10% FCS or 10% LPDS. Cell nuclei were counterstained with DAPI (blue). Representative images are shown. Scale bar: 10 µm.

Besides the increase in SA-β-Gal activity, further studies showed that a loss of the *LMNB1* gene and lamin B1 protein expression is associated with cellular senescence [21,22,23]. No changes in the *LMNB1* gene as well as in lamin B1 protein expression were observed for PXE fibroblasts compared to NHDF when cultivated in a medium with 10% FCS (Figure 2A,B). On the application of 10% LPDS, a significant decrease of *LMNB1* gene expression was detected for PXE fibroblasts compared to NHDF (control: 0.66 ± 0.04, PXE: 0.27 ± 0.03; *p* ≤ 0.001). Accordingly, a significant decrease for *LMNB1* gene expression could be seen for PXE fibroblasts cultivated in 10% LPDS compared to PXE fibroblasts cultivated in 10% FCS (PXE in 10% FCS: 0.70 ± 0.12, PXE in 10% LPDS: 0.27 ± 0.03; *p* ≤ 0.01). However, no significant changes were observed for lamin B1 protein level for PXE fibroblasts compared to NHDF under these conditions.

### 2.3. Alteration in Gene Expression of the Cyclin-Dependent Kinase Inhibitors (CDKI) p21 and p27

**Figure 3:**
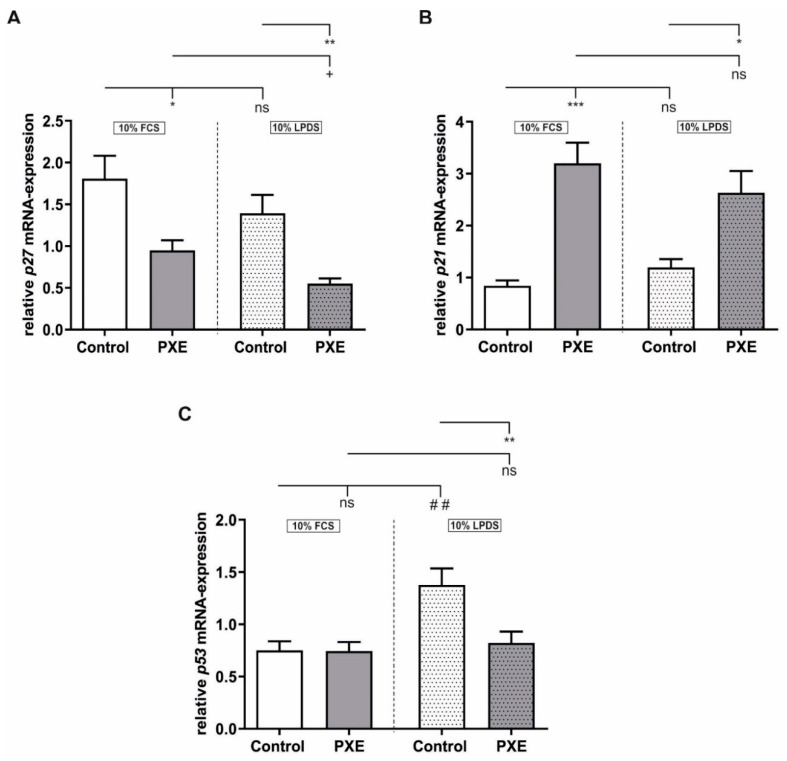
Relative *p27, p21* und *p53* mRNA-expression of PXE fibroblasts and NHDF. Fibroblasts were cultivated for 24 h in medium with 10% FCS or 10% LPDS. (**A**) Relative *p27* mRNA-expression of PXE fibroblasts (gray, *n* = 3) and NHDF (white, *n* = 3). (**B**) Relative *p21* mRNA-expression of PXE fibroblasts (gray, *n* = 3) and NHDF (white, *n* = 3). (**C**) Relative *p53* mRNA-expression of PXE fibroblasts (gray, *n* = 3) and NHDF (white, *n* = 3). Data are shown as mean ± SEM. Control/ PXE: * *p* ≤ 0.05; ** *p* ≤ 0.01; *** *p* ≤ 0.001; ns *p* > 0.05. 10% FCS/ 10% LPDS: + *p* ≤ 0.05; ## *p* ≤ 0.01; ns *p* > 0.05.

The initiation of cellular senescence is dependent on different CDKIs, which suppress the phosphorylation of the retinoblastoma protein (pRB) [24,25]. Thus, we analyzed the CDKIs *p27*, *p21* and *p53*. As seen in Figure 3A a significant decrease in *p27* gene expression was detected for PXE fibroblasts compared to NHDF when cultivated in 10% FCS (control: 1.81 ± 0.27, PXE: 0.95 ± 0.12; *p* ≤ 0.05) as well as in 10% LPDS (control: 1.39 ± 0.22, PXE: 0.55 ± 0.06; *p* ≤ 0.01). Additionally, a significant downregulation of *p27* gene expression was observed for PXE fibroblasts cultivated in 10% LPDS when compared to PXE fibroblasts cultivated in 10% FCS (PXE in 10% FCS: 0.95 ± 0.12, PXE in 10% LPDS: 0.55 ± 0.06; *p* ≤ 0.05). For *p21* mRNA expression, a significant increase was detected in PXE fibroblasts compared to NHDF when cultivated in medium with 10% FCS (control: 0.84 ± 0.10, PXE: 3.20 ± 0.40; *p* ≤ 0.001) as well as in medium with 10% LPDS (control: 1.20 ± 0.16; PXE: 2.63 ± 0.42; *p* ≤ 0.05). No significant changes were observed between the different media. As seen in Figure 3C, no significant changes in *p53* gene expression were seen for PXE fibroblasts compared to NHDF when cultivated in a medium with 10% FCS. A significant increase in *p53* gene expression was observed for control fibroblasts cultivated in 10% LPDS compared to control fibroblasts when cultivated in 10% FCS (control in 10% FCS: 0.75 ± 0.09, control in 10% LPDS: 1.38 ± 0.16; *p* ≤ 0.01). In accordance with this, a significant decrease was detected for PXE fibroblasts cultivated in 10% LPDS when compared to NHDF also cultivated in 10% LPDS (control: 1.38 ± 0.16, PXE: 0.82 ± 0.11; *p* ≤ 0.01). No differences in *p53* gene expression were detected for PXE fibroblasts between the different media. We further analyzed different factors of cell cycle control. As seen in Table 1, no significant changes in protein concentration for the listed proteins of cell cycle control, which are measured by cell cycle antibody array, were detected for PXE fibroblasts compared to controls when cultivated in 10% LPDS.

### 2.4. Strong Increase in Interleukin 6 (IL6) Gene and Protein Expression

**Figure 4:**
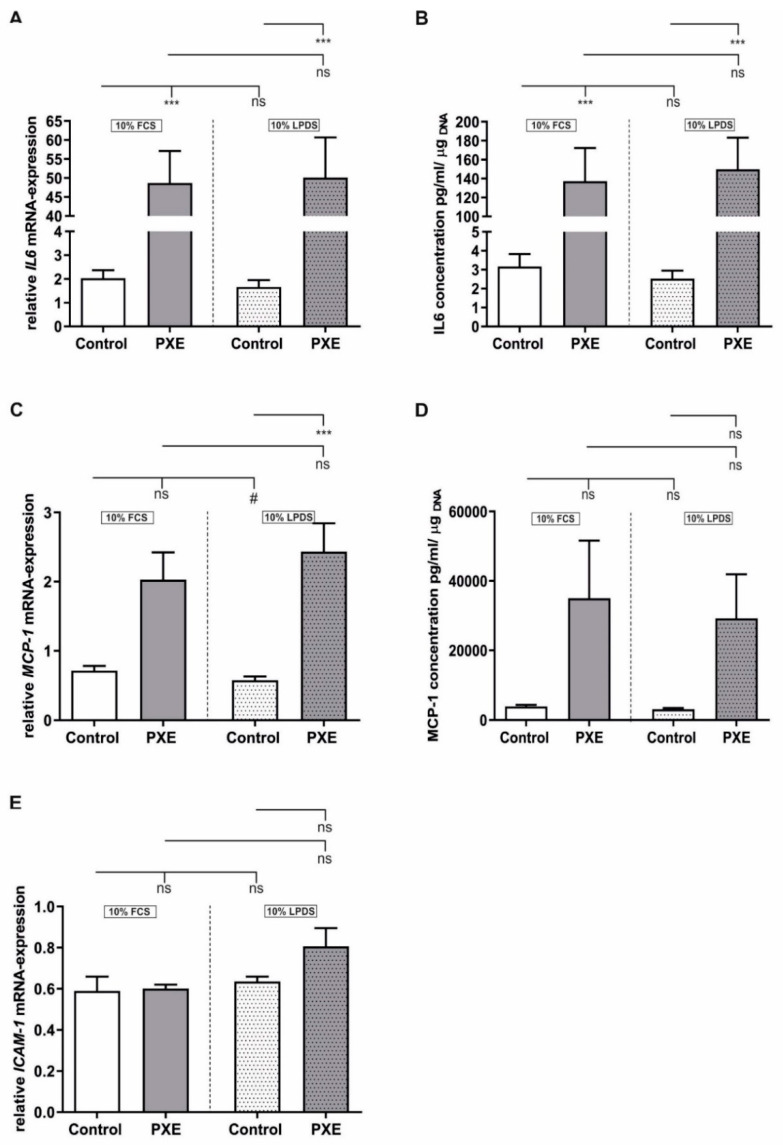
Relative mRNA-expression und protein expression of proinflammatory factors in PXE fibroblasts and NHDF. Fibroblasts were cultivated for 21 d in medium with 10% FCS or 10% LPDS. (**A**) Relative *IL6* mRNA-expression of PXE fibroblasts (gray, *n* = 3) and NHDF (white, *n* = 3). (**B**) IL6 protein concentration in supernatants of PXE fibroblasts (gray, *n* = 3) and NHDF (white, *n* = 3). (**C**) Relative *MCP1* mRNA-expression of PXE fibroblasts (gray, *n* = 3) and NHDF (white, *n* = 3). (**D**) MCP1 protein concentration in supernatants of PXE fibroblasts (gray, *n* = 3) and NHDF (white, *n* = 3). (**E**) Relative *ICAM1* mRNA-expression of PXE fibroblasts (gray, *n* = 3) and NHDF (white, *n* = 3). Data are shown as mean ± SEM. Control/PXE: *** *p* ≤ 0.001; ns *p* > 0.05. 10% FCS/10% LPDS: # *p* ≤ 0.05; ns *p* > 0.05.

A well-known characteristic of senescent cells is the development of a senescence associated secretory phenotype (SASP) consisting of different proinflammatory factors like IL6 or monocyte chemoattractant protein 1 (MCP1). For the *IL6* gene expression (Figure 4A) and for protein concentration in supernatants (Figure 4B), a strong induction was observed for PXE fibroblasts compared to NHDF when cultivated in medium with 10% FCS (mRNA; control: 2.03 ± 0.34; PXE: 48.68 ± 8.46; *p* ≤ 0.001; Protein; control: 3.17 ± 0.65; PXE: 137.10 ± 35.28; *p* ≥ 0.001) as wells as when cultivated in medium with 10% LPDS (mRNA; control: 1.66 ± 0.29, PXE: 50.13 ± 10.56; *p* ≤ 0.001; Protein; control: 2.53 ± 0.43; PXE: 149.8 ± 33.47; *p* ≤ 0.001). As seen in Figure 4C,D *MCP1* gene expression and protein concentration in supernatants were increased in PXE fibroblasts compared to NHDF in both media, but only the upregulation of *MCP1* gene expression in PXE fibroblasts compared to NHDF when cultivated in medium with 10% LPDS reached statistical significance (control: 0.58 ± 0.06, PXE: 2.43 ± 0.41; *p* ≤ 0.001). Furthermore, *MCP1* gene expression showed a significant decrease for control fibroblasts cultivated in 10% LPDS compared to control fibroblast cultivated in 10% FCS (control in 10% FCS: 0.72 ± 0.06; control in 10% LPDS: 0.58 ± 0.06; *p* ≤ 0.05). No significant differences were observed for *Intercellular adhesion molecule 1* (*ICAM1)* gene expression.

## 3. Discussion

PXE is a rare autosomal-recessive disorder caused by mutations in the *ABCC6* gene, which lead to a deficiency in the corresponding ABC-transporter protein [1]. Although several molecular characteristics of PXE direct towards a potential association with premature cellular senescence and accelerated aging processes [5,6,7], little is known, especially about the potential involvement of cells of peripheral tissues. Because of this, we analyzed different senescence-associated factors in primary human dermal fibroblasts of PXE patients to further evaluate premature aging processes in peripheral tissues. As previous studies showed that lipid metabolism might play an important role in PXE pathogenesis [20], fibroblasts were cultured in a medium supplemented with 10% FCS or 10% LPDS. LPDS was especially applied to reveal effects that would be otherwise potentially covert by an excess of exogenous lipoproteins when conventional FCS is used.

The first indications pointing towards cellular senescence are morphological changes. Fibroblasts undergo cellular senescence change from an elongated shape to an enlarged and flattened irregular cell morphology [26,27]. These morphological changes were also seen here for PXE fibroblasts compared to NHDF. Another senescence-associated biomarker is the SA-β-Gal activity, which is increased in senescent cells. β-galactosidase is a lysosomal protein with a pH optimum between 4.0 and 4.5. In the case of cellular senescence, the SA-β-Gal activity raises to a degree where it can even be detected at pH 6.0 [28]. Until now, only a study of Miglionico et al. could show increased cellular senescence in HepG2 cells carrying an *ABCC6* knockdown [18]. In this study, we were now able to detect a significant increase in SA-β-Gal activity in primary human dermal fibroblasts of PXE patients in vitro, which potentially indicates a direct influence of *ABCC6* deficiency on affected peripheral tissues supporting the cellular hypothesis of PXE pathomechanism. As SA-β-Gal is lysosomal β-Gal, several studies refer to increased SA-β-Gal activity to an increased lysosomal protein concentration or activity of ß-galactosidase or to an accumulation of lysosomes [29,30]. Therefore, we detected protein levels of LAMP1, a ubiquitous lysosomal membrane protein, to evaluate a possible accumulation of lysosomes. As we could not detect significant differences in LAMP1 between PXE fibroblasts and NHDF neither in medium with 10% FCS nor in medium with 10% LPDS, the increased SA-β-Gal activity in PXE fibroblasts is, therefore, probably not associated with an accumulation of lysosomes, but with increased lysosomal activity or an increased β-galactosidase protein concentration within the lysosomes. Apart from this, we observed a decreased SA-β-Gal activity in PXE fibroblasts when cultivated in 10% LPDS compared to PXE fibroblasts cultivated in 10% FCS, but an increase in LAMP1 on the application of 10% LPDS compared to the cultivation in 10% FCS. This shows that the withdrawal of lipoproteins results in an accumulation of lysosomes to probably control energy balance, but the SA-β-Gal protein is less active on lipoprotein depletion, although the reason for this remains unclear.

Apart from an increase in SA-β-Gal activity, cellular senescence is also associated with a loss in lamin B1 expression [21,22,23]. In this study, no significant differences in *LMNB1* mRNA-expression between PXE fibroblasts and NHDF were detected when cultivated in 10% FCS. However, under lipoprotein depleted conditions, a significant decrease of *LMNB1* gene expression was measured for PXE fibroblasts compared to NHDF under the same conditions as wells as compared to PXE fibroblasts cultivated in 10% FCS. A previous study showed that the loss of lamin B1 is associated with the stimulation of p53 and the pRB [21]. Thus, lamin B1 expression is controlled by the transcription factor E2F, which is bound to hypophosphorylated pRB. Phosphorylation of pRB by different cyclin-dependent kinases (CDK) leads to E2F release and initiation of *LMNB1* expression. CDKs are controlled by different CDKI, which suppress pRB phosphorylation and, thus, the release of E2F. If pRB remains hypophosphorylated because of CDK inhibition, the result is a permanent cell cycle arrest and a reduction in E2F-dependent proteins like lamin B1 [22,24,25]. For PXE fibroblasts, we found no significant changes in E2F or cyclin protein level compared to NHDF when cultivated in 10% LPDS. However, we found a significant increase in *p21* gene expression for PXE fibroblasts compared to NHDF when cultivated in 10% FCS as well as in 10% LPDS. No simultaneous induction of *p53* gene expression was observed in PXE fibroblasts, which leads to the assumption of a p53-independent p21-mediated mechanism for the induction of cellular senescence in PXE fibroblasts. Additionally, the stable *p53* gene expression raises the question of whether the reduction in *LMNB1* mRNA-expression seen for PXE fibroblasts is directly dependent on the p53/pRB pathway. It could be, thus, rather a result of the lack of lipoproteins by cultivation in 10% LPDS as lamin B1 maturation is strongly dependent on prenylation processes, which are themselves dependent on cholesterol biosynthesis [31]. Previous studies showed that cholesterol biosynthesis is strongly induced in PXE fibroblasts, especially when cultivated in 10% LPDS [20]. *p21* could be further upregulated by binding of the transcription factors signal transducer and activator of transcription (STAT)1 or STAT3 in a p53-independent manner [32]. STAT3 is part of the Janus kinase (JAK)–STAT signal pathway that can be activated by small GTPases, which are in turn dependent on prenylation processes as well [33,34,35,36,37]. Nevertheless, we found no significant increase in p21 protein level, which raises the question of whether p21 is indeed the trigger of cellular senescence in PXE fibroblasts. In contrast to *p21*, we detected a significant decrease in *p27* gene expression for PXE fibroblasts in both media. An increased *p27* gene expression is associated with a temporary proliferation arrest due to contact inhibition [38,39]. As PXE fibroblasts show increased cellular senescence compared to NHDF cell–cell contact and, thus, cell density in cultures of PXE fibroblasts is probably lower. Consequently, induction of *p27* gene expression could probably not be detected for PXE fibroblasts.

However, another well-known characteristic of senescent cells is the expression and secretion of a SASP. We recently showed aberrant gene expression of the secreted proteins insulin-like growth factor-binding protein 3 (IGFBP3) as well as growth differentiation factor 11 for PXE fibroblasts indicating alterations of the secretome probably due to premature aging. In the same study, we additionally have seen a clear sexual dimorphism with decreased IGFBP3 expression in PXE fibroblasts from male donors and no differences in PXE fibroblasts from female donors compared to the appropriate control fibroblasts [40]. Here, we detected a drastically increased *IL6* mRNA-expression as well as IL6 protein concentration in cell culture supernatants for PXE fibroblasts compared to NHDF when cultivated in medium with 10% FCS as well as in medium with 10% LPDS. However, we did not see a considerable significant sexual dimorphism either for IL6 or for any of the other tested targets in this study. This indicates that the previously observed sexual dimorphism probably only applies to specific factors in the case of PXE. Nevertheless, IL6 is a prominent component of a SASP, and it is known that IL6 can also activate STAT3 and, therefore, induce the JAK–STAT signal pathway. As a consequence, IL6 may stimulate its own expression in an autocrine manner and potentially participates in the induction of p21 expression and, thus, cellular senescence [32,36,41]. We further detected an increase in *MCP1* gene expression and MCP1 protein concentration in supernatants of PXE fibroblasts compared to NHDF in both media. Several previous studies already showed that IL6 could stimulate MCP1 expression in various cell types [42,43,44]. It was further shown that MCP1 could stimulate the β-galactosidase activity and expression of CDK inhibitors in human keratinocytes [45], which matches the present results and indicates a potential paracrine effect that may trigger cellular senescence in PXE fibroblasts. Besides MCP1, previous studies showed that IL6 can also stimulate ICAM1 expression and secretion and that gene expression of *ICAM1* is increased in senescent fibroblasts [46,47]. It is further known that increased concentrations of ICAM1 can be found in the serum of PXE patients as wells as in supernatants of *ABCC6* deficient C3H macrophages [48,49]. Although we detected a strong increase in *IL6* gene expression and protein concentration in supernatants of PXE fibroblasts, we did not see any significant increase in *ICAM1* gene expression. A previous study further showed that induction of ICAM1 expression is associated with an increase in p53 [50], which would explain the missing upregulation of *ICAM1* in the case of PXE fibroblasts.

**Figure 5:**
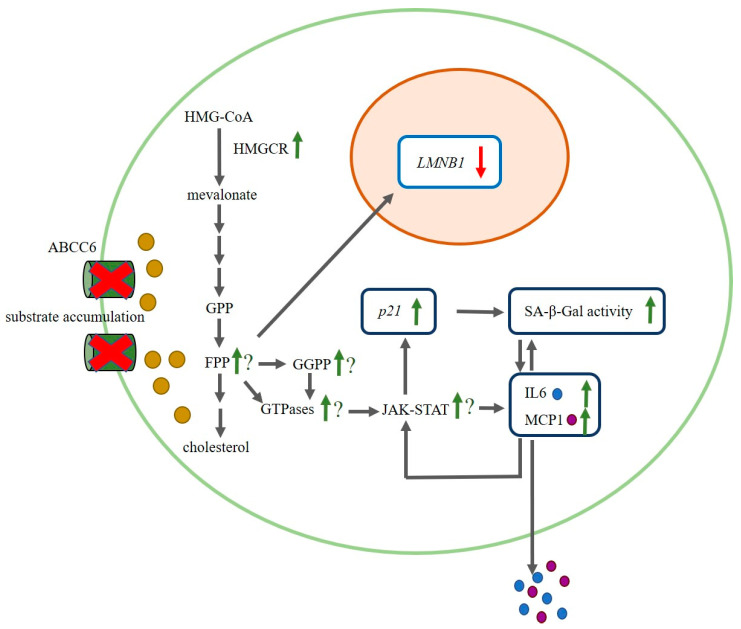
Suggested altered cellular pathways and their potential connection in PXE. The induction of cholesterol biosynthesis could lead to an increase in prenylation of small GTPases. Small GTPases are responsible for the activation of STAT3 and the Janus kinase (JAK)–STAT signal pathway. STAT3 could, thus, further upregulate p21, which in turn could induce cellular senescence and SA-β-Gal activity. Due to the development of a proinflammatory SASP, IL6 and MCP1 expression would increase and could further contribute to the induction of STAT3 and the JAK–STAT signal pathway. In addition to this, the induction of the cholesterol biosynthesis could lead to a reduction of *LMNB1* gene expression under lipoprotein depleted conditions as a compensatory effect *ABCC6*: *ATP-binding cassette sub-family C member 6*; FPP: farnesylpyrophosphate; GPP: geranylpyrophasphate; GGPP: geranylgeranylpyrophosphate; HMG-CoA: 3-hydroxy-3-methyl-glutaryl-coenzyme A; HMGCR: 3-hydroxy-3-methyl-glutaryl-coenzyme A reductase; IL6: interleukin 6; JAK–STAT: Janus kinase–signal transducer and activator of transcription; *LMNB1*: lamin B1; MCP1: monocyte chemoattractant protein-1; SA-β-Gal activity: senescence-associated-β-galactosidase activity.

This is the first study linking an *ABCC6* deficiency in primary human dermal fibroblasts of PXE patients to premature cellular senescence. The suggested altered cellular pathways and their potential connection in PXE are summarized in Figure 5. The further data point to a p21-triggered mechanism for the initiation of cellular senescence and the development of a proinflammatory SASP with drastically increased IL6 expression and secretion for PXE fibroblasts. However, because of potential high interindividual variability of factors like IL6 [51,52], further studies, e.g., with control fibroblasts from far older individuals or control fibroblasts of high passages with increased replicative senescence, need to be done to be able to better relate the severity of the PXE phenotype detected in this study to an actually aged phenotype. Additionally, studies to evaluate potentially involved mechanisms like the JAK–STAT signal pathway would give further insights into PXE pathomechanism.

## 4. Materials and Methods

### 4.1. Experimental Design

The study was designed to evaluate potential premature aging processes in PXE on a cellular level using primary human dermal fibroblasts of PXE patients and healthy controls. Thus, fibroblasts were cultured in a medium supplemented with either 10% FCS or 10% LPDS. As previous studies showed that the cholesterol biosynthesis might play a crucial role in PXE pathogenesis [20], LPDS was used to reveal effects that would be otherwise maybe covert by an excess of exogenous lipoproteins applied by conventional FCS. Premature aging processes in PXE fibroblasts were determined by evaluating the SA-β-Gal activity and LAMP1 protein level as wells as *LMNB1* gene and lamin B1 protein level. Furthermore, *p27*, *p53,* as well as *p21* mRNA-expression, were examined. Additionally, protein expression of several factors of the cell cycle control was determined by a Cell Cycle Antibody Array. As senescent cells are characterized by a proinflammatory secretory phenotype, gene expression and protein concentrations in cell culture supernatants of factors like IL6, MCP1, as well as ICAM1 were measured.

### 4.2. Cell Culture

NHDF were purchased from Coriell Institute for Medical Research (Camden, NJ, USA). Dermal fibroblasts from PXE patients were isolated from skin biopsies [53]. Information about fibroblasts characteristics with the appropriate available Phenodex scores, according to Legrand et al. [3], are listed in Table 2. All patients and controls gave their informed consent for participation in the study. The study was conducted in accordance with the Declaration of Helsinki and approved by the Ethics Committee of the HDZ NRW, Department of Medicine, Ruhr University of Bochum (registry no. 32/2008, Approval date is 3 November 2008).

Fibroblasts were cultivated in Dulbecco’s modified essential medium (DMEM, Gibco, Thermo Fisher Scientific, Waltham, MA, USA) supplemented with 10% FCS (Biowest Nuaillé, France), 2% l-glutamine (200 mM) (PAN Biotech, Aidenbach, Germany) and 1% antibiotic/antimycotic solution (PAA Laboratories, Pasching, Austria). When reaching confluency, fibroblasts were subcultured.

Fibroblasts between passages 9 and 12 were used. Biological samples were prepared in triplicates. For all experiments, cells were seeded in a final cell density of 177 cells/mm^2^. Fibroblasts were cultivated for 24 h in 10% FCS. The next day, the medium was changed to fresh 10% FCS or 10% LPDS for an additional 24 h, 72 h or 21 days. In the case of 21 days, the medium was changed every 2–3 days.

### 4.3. Delipidation of FCS

LPDS was prepared, according to Gibson et al. [54]. 1 g Cab-o-sil (silicic acid powder) was mixed with 50 mL FCS and incubated at 4 °C overnight. The next day, the mixture was centrifuged for 1 h at 4 °C, and 5000× *g* and the clarified supernatant was transferred to a new tube and stored at −20 °C. For the preparation of fresh medium, LPDS was sterile filtered (0.2 µm). After delipidation, free cholesterol is reduced by about 78%, LDL by about 95% and HDL by about 57%, whereas triglyceride concentrations remained unchanged.

### 4.4. Nucleic Acid Isolation

For RNA isolation, the NucleoSpin RNA Kit (Macherey–Nagel, Düren, Germany) was used. DNA isolation for normalization of immunoassay measurements was performed using the NucleoSpin Blood extraction Kit (Macherey–Nagel, Düren, Germany). Procedures were performed according to the manufacturer’s instructions.

### 4.5. Bicinchoninic Acid Assay

Bicinchoninic acid (BCA) assay was performed for the determination of protein concentration in cell lysates. For analysis, 25 µL cell lysate was mixed with 200 µL copper (II) sulfate solution/ bicinchoninic acid (1:50). A standard curve was prepared using a serial dilution of bovine serum albumin (stock: 1 mg/mL). The reaction mixture was incubated for 30 min at 37 °C. Absorption was measured at 562 nm using a Tecan Reader infinite 200 Pro (Tecan, Männedorf, Switzerland). Protein concentrations were used for the normalization of quantitative β-galactosidase activity measurements.

### 4.6. Gene Expression Analysis

For cDNA synthesis, 1 µg RNA was transcribed into cDNA using SuperScript II Reverse Transcriptase (Thermo Fisher Scientific, Waltham, MA, USA). For *p27*, *p53*, *p21, LMNB1, IL6, MCP1* and *ICAM1* 2.5 μL cDNA (1:10), 0.25 μL forward and reverse primer (Biomers, Ulm, Germany), 2.0 μL water and 5.0 μL LightCycler 480 SYBR Green I Master reaction mixture (Roche, Penzberg, Germany) was mixed for each measurement. After an initial incubation for 5 min at 95 °C, the qPCR protocol involves 45 cycles of denaturation (95 °C, 10 s), annealing (specific annealing temperature, 15 s) and elongation (72 °C, 20 s). Relative mRNA expression of measured targets was normalized to relative *β-actin (β-ACTIN)*, *glyceraldehyde-3-phosphate-dehydrogenase (GAPDH)* and *β2-microglobulin (β2M)* mRNA expression. After amplification, melting curve analysis was performed. Measurements of gene expression were conducted using LightCycler 480 (Roche, Penzberg, Germany). Relative mRNA expression was calculated using the delta-delta Ct method considering PCR efficiency. Technical triplicates were done for each biological sample. Sequences of forward and reverse primers can be found in Table 3.

### 4.7. Immunofluorescence Microscopy

For immunofluorescence experiments, coverslips (Ø18 mm) were placed into a 12-well plate and coated with 5 µg/cm^2^ rat collagen (ibidi, Gräfelfing, Germany). Fibroblasts were seeded with the previously stated cell density and incubated for 24 h in 10% FCS. Afterward, the medium was changed by fresh 10% FCS or 10% LPDS for a further 72 h.

After the stated time of cultivation, fibroblasts were washed with phosphate-buffered saline (PBS; Gibco, Thermo Fisher Scientific, Waltham, MA, USA). Fixation and permeabilization of cells were done by incubating with acetone:methanol (1:1) for 10 min at room temperature. Afterward, cells were washed again twice with PBS and incubated for 1 h in 1% bovine serum albumin (BSA) diluted in PBS to block unspecific binding sites. After two further washing steps, fibroblasts were incubated with primary antibodies for 2 h at room temperature. Primary antibodies used included rabbit anti-lamin B1 (ab16046; Abcam (Cambridge, UK); 1:1000) and mouse anti-LAMP1 (ab25630; Abcam (Cambridge, UK); 1:50). After another two washes, fibroblasts were incubated for 1 h with the secondary antibody at room temperature under the exclusion of light. For the secondary antibody, a FITC-conjugated goat anti-rabbit (1:75) (Jackson Immuno Research, West Grove, PA, USA) was used. Antibodies were diluted in 0.1% BSA in PBS. Fibroblasts were washed again twice and were counterstained with DAPI. The counterstaining was followed by another three washes with PBS. Coverslips were mounted with ProLong Diamond Antifade Mountant (Thermo Fisher Scientific, Waltham, MA, USA). Immunofluorescence images were captured by fluorescence microscopy using the microscope TE2000-S (Nikon GmbH, Düsseldorf, Germany) or confocal laser scanning microscopy TCS SP8 system (Leica Microsystems, Wetzlar, Germany). For confocal laser scanning microscopy, FITC was excited at 488 nm, and emission was detected between 493 and 557 nm.

### 4.8. Qualitative Senescence Assay

For the evaluation of the number of senescent fibroblasts in culture, the β-galactosidase activity was determined, according to Dimri et al. [28]. In brief, fibroblasts were seeded as previously stated in 6-well plates and cultivated in 10% FCS for 24 h. Afterward, the medium was replaced by either medium supplemented with 10% FCS or 10% LPDS and cultivated for another 72 h. After the stated time of growth, the medium was removed, and fibroblasts were washed twice with PBS. Fibroblasts were fixed with 2 mL 3% formaldehyde for 3–5 min at room temperature. Afterward, two further washing steps were performed and followed by application of the substrate solution (0.2 M sodium phosphate, 0.1 M citric acid, 5 mM potassium ferrocyanide (trihydrate), 5 mM potassium ferrocyanide, 150 mM NaCl, two mM MgCl_2_ (hexahydrate), 1 mg/mL 5-bromo-4-chloro-3-indolyl-β-d-galactopyranoside, pH 6.0). Fibroblasts were incubated overnight at 37 °C without CO_2_. The next day, images were captured using the microscope TE2000-S (Nikon GmbH, Düsseldorf, Germany).

### 4.9. Quantitative Senescence Assay

Quantitative determination of β-galactosidase activity was conducted according to a previous protocol of Gary and Kindell [55]. In brief, fibroblasts were seeded according to previously mentioned instructions in 60 mm cell culture dishes and incubated for 24 h in 10% FCS. Afterward, the medium was changed with fresh medium with 10% FCS or 10% LPDS and fibroblasts were cultivated for another 72 h. After this, the medium was removed, and fibroblasts were washed four times with PBS. 300 µL lysis buffer (0.2 M sodium phosphate, 0.1 M citric acid, 5 mM CHAPS, 0.5 mM benzamidine, 0.25 mM PMSF, pH 6.0) was applied onto the cell layer and fibroblasts were detached using a cell scraper. Lysates were centrifuged for 5 min at 12,000× *g*. 50 µL of the clarified supernatant was used for the BCA assay. 100 µL of the supernatant was mixed with 100 µL of reaction buffer (0.2 M sodium phosphate, 0.1 M citric acid, 300 mM NaCl, 10 mM β-mercaptoethanol, 4 mM MgCl_2_, 1.7 mM 4-methylumbelliferyl-β-d-galactopyranoside, pH 6.0) and incubated for 1 h at 37 °C. After incubation, 50 µL of the reaction mixture was added to 150 µL of a 400 mM sodium carbonate solution to stop the reaction. 150 µL of the mixture was added to a black 96-well plate and measured using a Tecan Reader Infinite 200 PRO (excitation: 360 nm, emission: 465 nm). Results were normalized to protein concentrations of cell lysates.

### 4.10. Immunoassays for Evaluation of SASP Factors in Cell Culture Supernatants

For the determination of protein concentrations of MCP1 as well as ICAM1 in cell culture supernatants, commercially available ELISA Kits (R&D Systems, Abingdon, UK) were used. Measurements of IL6 concentrations in cell culture supernatants were conducted using the immunoanalyzer Cobas e411 (Roche, Basel, Switzerland). Results were normalized to DNA content.

### 4.11. Cell Cycle Antibody Array

For the antibody array (Full Moon BioSystems, Sunnyvale, CA, USA), cells were seeded in 60 mm cell culture dishes with the previously stated cell density and incubated for 24 h in 10% FCS. To achieve the required protein concentration, eight biological replicates were performed. The next day, the medium was changed to 10% LDPS and cells were cultivated for a further 72 h. After the stated time of growth, cells were washed four times with PBS, and 150 µL RIPA buffer (Thermo Fisher, Waltham, MA, USA) was applied to the first of the eight biological replicates. Cells were detached using a cell scraper. The cell lysate of the first biological replicate was afterward transferred to the second biological replicate, and cells were again detached with a cell scraper before the whole cell lysate is transferred to the third biological replicate. The procedure was repeated until the eighth biological replicate. The protein concentrations of the resulting cell lysates were determined by BCA assay. Afterward, all cell lysates were diluted to a concentration of 3 mg/mL. Cell lysates of PXE fibroblasts were mixed, and cell lysates of NHDF were mixed. The cell cycle antibody array was conducted by the manufacturer according to their instructions for both samples. In brief, proteins were biotinylated by incubation of the cell lysates with a biotin/DMF solution (10 μg/μL) for 2 h at room temperature. Antibody array slides were incubated with blocking solution for 40 min at room temperature to block unspecific binding sides. Afterward, the blocking solution was removed. Each biotinylated protein sample was applied onto one antibody array slide and was incubated for 2 h at room temperature. After incubation, slides were washed several times with deionized water and incubated with a Cy3-streptavidin solution (1 mg/mL) for 45 min at room temperature under the exclusion of light. After several washing steps, the detection was performed with an array scanner.

### 4.12. Statistical Analysis

Data of gene expression analyses and protein concentrations in cell culture supernatants are shown as mean ± standard error (SEM). As a statistical software GraphPad Prism 5.0 (GraphPad, San Diego, CA, USA) was used. For analyses, the nonparametric two-tailed Mann–Whitney U test was performed. *p*-values of 0.05 or less were considered statistically significant.

## Figures and Tables

**Table 1:** Cell cycle antibody array data. Shown are the mean signal intensities of spots and the ratio of PXE and control. PXE/control ratios of 2 or 0.5 are considered statistically significant.

Array Position	Protein	Mean Signal Intensity of Spots		Ratio PXE/Control
		Control	PXE	
Block 1, No. 5	Cyclin B1	585	492	0.84
Block 1, No. 6	Cyclin C	284	260	0.92
Block 1, No. 7	Cyclin E	2237	1715	0.77
Block 1, No. 8	Cdk3	964	887	0.92
Block 1, No. 9	Cdk8	560	514	0.92
Block 1, No. 10	CDC37	284	262	0.92
Block 1, No. 18	APC11	401	380	0.95
Block 2 No. 8	Cdk1/p34^cdc2^	901	772	0.86
Block 2, No. 9	Cyclin D1	259	228	0.88
Block 2, No. 14	p21^WAF1^	240	262	1.09
Block 2, No. 17	E2F-2	302	335	1.11
Block 3, No. 10	p130^cas^	284	332	1.17
Block 4, No. 5	Ki67	304	257	0.85
Block 4, No. 6	Chk1	518	424	0.82
Block 4, No. 7	14.3.3, Pan	682	866	1.27
Block 4, No. 8	Cullin-3 (CUL-3)	4227	3821	0.90
Block 4, No. 12	GSK3b	1733	1153	0.67
Block 4, No. 13	p19^ARF^	2247	1994	0.89
Block 4, No. 14	p57^Kip2^	310	258	0.83

**Table 2:** Characteristics of fibroblasts used.

Sample ID	Gender	Age ^1^	Biopsy Source	*ABCC6* Genotype ^2^		Genotype Status	Phenodex Score ^3^
**PXE patients**							
P3M ^a^	male	57	Neck	c.3421C>T (p.Arg1141*)	c.3883-6G>A (SSM)	cht	V2; C0
P128M ^a^	male	51	Neck	c.3769_3770insC (p.L1259fsX1277)	c.3769_3770insC (p.L1259fsX1277)	hm	S2; E2; G0; C1
P255F ^a^	female	48	Arm	c.3421C>T (p.Arg1141*)	c.2787+1G>T	cht	S3; E2; G0; C0
**Healthy controls**							
M57A ^b^ (AG13145)	male	57	Arm	-	-	wt	Not applicable
M52A ^b^ (AG11482)	male	52	Arm	-	-	wt	Not applicable
F48A ^b^ (AG14284)	female	48	Arm	-	-	wt	Not applicable

hm, homozygous; cht, compound heterozygous; wt, wild type; SSM, splice site mutation ^a^ Fibroblasts isolated from skin biopsies [53]. ^b^ Fibroblasts purchased from Coriell Institute for Medical Research (Camden, NJ, USA). ^1^ Age in years. ^2^ Nucleotide numbering refers to the cDNA sequence with the A of the ATG translation initiation start site as nucleotide +1 (GenBank accession number NM_001171.2). ^3^ adapted from the Phenodex score (an internationally standardized scoring system for uniform evaluation of PXE cases) according to Legrand et al. [3]. S: skin; E: eye; G; gastrointestinal; V: vascular; C: cardiac.

**Table 3:** Primer sequences used for quantitative real-time PCR.

Gene	Protein	5′-3′Sequence	Reference ^1^	Annealing Temperature (°C)	Efficiency
*β-ACTIN* *beta-Actin*	β-Actin	CGCGAGAAGATGACCC ATTGCCAATGGTGATGAC	NM_001101	59	2.0
*GAPDH* *glycerinaldehyd-3-phosphat-dehydrogenase*	GAPDH	AGGTCGGAGTCAACGGAT TCCTGGAAGATGGTGATG	NM_002046	59	1.8
*β2M* *beta-2-* *microglobulin*	β2M	TGTGCTCGCGCTACTCTCTCTT CGGATGGATGAAACCCAGACA	NM_004048	59	2.0
*ICAM1* *Intercellular adhesion molecule 1*	ICAM1	ACCATCTACAGCTTTCCGGC CAATCCCTCTCGTCCAGTCG	NM_000201.3	63	1.9
*IL6* *Interleukin 6*	IL6	ACAGCCACTCACCTCTTCAG GTGCCTCTTTGCTGCTTTCAC	NM 000600.4	63	1.9
*LMNB1* *Lamin B1*	Lamin B1	GCAGACTTACCATGCCAAAC TCCCTTATTTCCGCCATCTC	NM 005573.3	63	1.9
*MCP1* *Monocyte chemotactic* *Protein 1*	MCP1	CTTCTGTGCCTGCTGCTCATA GGACACTTGTCGCTGGTGATT	NM 002982.3	66	2.0
*p21/CDKN1A* *cyclin-dependent kinase inhibitor 1A*	p21	GCAGACCAGCATGACAGATTTC ACCTCCGGGAGAGAGGAAAA	NM_000389.4	66	1.8
*p27/CDKN1B* *Cyclin-dependent kinase inhibitor 1B*	p27	CAGCTTGCCCGAGTTCTACT AGAAGAATCGTCGGTTGCAGG	NM_004064.4	66	2.0
*p53* *tumor protein p53*	p53	AGATAGCGATGGTCTGGC TTGGGCAGTGCTCGCTTAGT	NM_000546.5	63	2.0

^1^ Accession numbers from reference sequences taken from GenBank are shown.

## Abbreviations

ABCC6	ATP-binding cassette sub-family C member 6
CDK	Cyclin-dependent kinase
CDKI	Cyclin-dependent kinase inhibitor
FCS	Fetal calf serum
HGPS	Hutchinson–Gilford progeria syndrome
ICAM1	Intercellular adhesion molecule 1
IL6	Interleukin 6
JAK	Janus Kinase
LAMP1	Lysosomal-associated membrane protein 1
LMNB1	Lamin B1
LPDS	Lipoprotein-deficient fetal calf serum
MCP1	Monocyte chemoattractant protein-1
MMP	Matrix metalloproteinase
NHDF	Normal human dermal fibroblasts
PPi	Pyrophosphate
pRB	Retinoblastoma protein
PXE	Pseudoxanthoma elasticum
SA-β-Gal	Senescence-associated β-galactosidase
SASP	Senescence-associated secretory phenotype
STAT	Signal transducer and activator of transcription
